# Placement of vena cava filter via percutaneous puncture of the great saphenous vein

**DOI:** 10.3892/etm.2013.1157

**Published:** 2013-06-14

**Authors:** YIQI JIN, DAYONG ZHOU, LEI CHEN, XIANCHEN HUANG, GUOXIONG XU, JIAN HUANG, LIMING SHEN

**Affiliations:** Department of Vascular Surgery, Affiliated Suzhou Hospital of Nanjing Medical University, Suzhou Municipal Hospital, Suzhou, Jiangsu 215002, P.R. China

**Keywords:** percutaneous puncture, great saphenous vein, vena cava filter

## Abstract

The aim of this study was to investigate the feasibility and safety of vena cava filter (VCF) placement via percutaneous puncture of the great saphenous vein (GSV) in the prevention of pulmonary embolisms. Using ultrasound positioning, VCF placement via percutaneous puncture of the GSV was performed on 12 patients with deep vein thrombosis (DVT) in the lower extremities. Transcatheter thrombolysis was conducted simultaneously. The postoperative filter position, puncture wound recovery and fluency of the GSV were observed. All filters were successfully released, with accurate positioning. No hematoma was observed at the puncture point during the perioperative period. In certain patients, local petechiae appeared around the puncture point during the thrombolysis period, which did not require special treatment. Re-examination using ultrasound revealed unobstructed blood flow in the GSV. VCF placement via percutaneous puncture of the GSV is a new filter placement method. The feasibility and safety of this method for the prevention of pulmonary embolisms has been demonstrated in a small number of sample cases.

## Introduction

The incidence of pulmonary embolism is increasing, and deep vein thrombosis (DVT) in the lower extremities is one of the main causes. It is reported that one-third of patients with an acute phase of DVT may suffer from acute pulmonary embolisms (1,2). Vena cava filter (VCF) placement is an effective measure for preventing pulmonary embolisms in DVT patients. It has the advantage of being a simple procedure, with minimal trauma and few complications, and has been widely applied clinically (3–6). As the outer sheaths of commercially available filters are becoming increasingly thinner, the traditional VCF placement method has changed from the percutaneous incision of a deep vein to Seldinger’s method (7,8), dominated by the percutaneous puncture of a deep vein. Currently, the placement pathways for VCFs are mainly the femoral and jugular veins (9), and the pathways of the subclavian, brachial and external jugular veins have also been reported (10,11). However, since a risk of complications arising from VCF placement via puncture of a deep vein remains, it is important to investigate safe and feasible puncture pathways for clinical use. The great saphenous vein (GSV), as a superficial vein, is not a traditional pathway for intracavitary therapy in lower extremity veins, but the puncture and incision via the superficial venous pathway has been reported (12,13). In the current study, using ultrasound positioning, the placement of a VCF via the percutaneous puncture of the GSV was performed on DVT patients, and the feasibility and safety of this method were investigated.

## Materials and methods

### General data

A total of 12 patients with DVT (5 males and 7 females) were enrolled in this study at the Affiliated Suzhou Hospital of Nanjing Medical University. The patients were aged between 48 and 87 years, with an average age of 51.5 years (Table I). The disease onset time was <1 week. All patients were diagnosed with unilateral DVT (left lateral, 9 cases; right lateral, 3 cases) using color Doppler ultrasound, venography and plasma D-dimer determination. According to the results of the venography, there were 4 central type, 4 peripheral type and 4 mixed type cases of DVT. VCFs were provided by Johnson & Johnson Co. (New Brunswick, NJ, USA). Trapease^®^ permanent filters were used to treat 4 patients, while the other 8 patients were treated with Optease^®^ temporary filters. THe present study was approved by the Ethics Committee of Affiliated Suzhou Hospital of Nanjing Medical University (Suzhou, China). Informed consent was obtained from the patient.

### Preoperative preparation

A preoperative routine blood test, blood coagulation test and other examinations were conducted on all patients. The inclusion criteria for VCF placement were as follows: definite pulmonary infarction symptoms or tendency, contraindication of anticoagulation, consideration of transcatheter thrombolysis, poor anticoagulation or re-thrombosis.

### Surgical methods

VCF placements via puncture of the unaffected lateral and affected lateral GSV were performed on 10 and 2 (central type) patients, respectively. The selected puncture point was located at the upper-middle segment of the inner thigh. After disinfection and local anesthesia, the thigh was ligatured using a tourniquet to temporarily block venous return and vein dilatation. Using color Doppler ultrasound positioning, puncture of the GSV was performed, and the gliding guide wire was placed, from the saphenous vein valve to the femoral vein and then on to the vena cava. The remaining surgical steps were the same as with a traditional filter placement method. The venography was conducted and once the outlet positions of bilateral renal veins were determined, the filter was released.

After releasing the filter, transcatheter thrombolysis in a deep vein by puncturing the jugular vein was undertaken in the majority of patients (14,15). For patients with thrombosis in the entirety of their lower extremities, transcatheter thrombolysis was conducted in the affected lateral femoral artery. For two patients with central type DVT, after puncturing the GSV, the catheter was placed directly into the thrombus for thrombolysis. The puncture wound of the GSV was treated with pressure bandaging.

### Postoperative treatment

After lying in a horizontal position for 6 h postoperatively, the patients were able to perform out-of-bed activities, with the exception of 2 cases where there was catheterization in the GSV. For patients undergoing thrombolytic and anticoagulant therapies, the coagulation change was monitored, and oozing ecchymosis at the puncture point and at the catheterization site was observed every day. The thrombolytic situation was monitored by venography to enable adjustment of the catheter position. The catheter was extubated 2 weeks after thrombolysis; furthermore, the temporary filters may be retrieved after thrombolysis.

## Results

**Figure 1.**
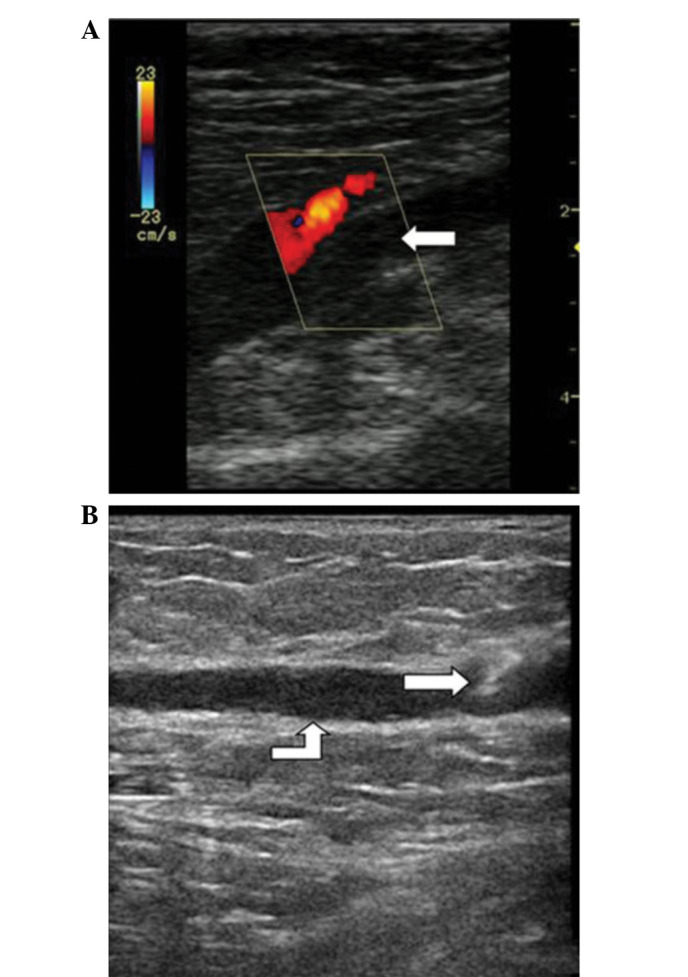
Color Doppler ultrasound of VCF placement. (A) deep vein thrombus (arrow); (B) puncture needle (straight arrow) and GSV (bent arrow). VCF, vena cava filter; GSV, great saphenous vein.

All filters were successfully released, using accurate ultra-sound positioning. In 6 out of 8 patients with temporary filter placement, filters were successfully retrieved within 3 weeks of surgery. A further two patients did not undergo retrieval due to economic reasons. The ultrasound-guided puncture was successfully conducted on all patients (Fig. 1). Throughout the perioperative period, there was no marked hematoma at the puncture point. For some patients, local petechiae appeared around the puncture point during the thrombolysis period, which did not require treatment. For all patients, re-examination with a color Doppler ultrasound revealed unobstructed blood flow in the GSV, with no back flow or thrombosis. Within 1 year of follow-up there had been no instances of pulmonary embolism.

## Discussion

At present, VCF placement via puncture of a deep vein (Seldinger’s method) is widely used in a clinical environment. This method has many advantages, including less trauma and fewer complications, and it is a simple surgery. However, puncture of a deep vein, particularly the femoral vein, has the following disadvantages: i) a hematoma is easily formed in anticoagulant and thrombolytic conditions, and it is difficult to detect and treat early due to its deep position. ii) There is a risk of local thrombosis at the puncture and catheterization site. iii) The femoral venous valve may be damaged. iv) Puncture is seldom successful for obese patients. v) The arteries may be mistakenly punctured, particularly for patients in whom the femoral vein is located on the dorsal side of the femoral artery. Additionally, the risk of hematoma and pseudoaneurysm formation will be aggravated during subsequent anticoagulant and thrombolytic therapy. vi) The puncture point is near the perineum, leading to problems with postoperative care (16,17).

However, puncture of the GSV may avoid the risks listed above. Most regions of the GSV are located at the superficial fascia layer. It is possible to detect bleeding or hematoma early and easily, and compression hemostasis is easily conducted. Due to shielding by the saphenofemoral valve, the inner wall and valve of the GSV to not readily become detached and enter the deep vein, even if the injury has induced secondary thrombosis. Therefore, the probability of deep vein valve injury is very low. The anatomical location of the GSV is superficial, with no accompanying artery. The puncture site is relatively flexible when using ultrasound positioning, and the iliac region may be avoided.

GSV puncturing has been performed by a number of physicians. For example, transcatheter thrombolysis and stent placement through the saphenous vein pathway have been conducted by interventional physicians with good results. Additionally, filter placement via GSV incision has also been performed, and the aforementioned advantages have been confirmed (18–24). Anatomical data indicate that the full diameter of a normal GSV is >2 mm and that in the thigh it is 3.2–4.0 mm (25). However, temporarily blocking superficial venous backflow may cause vein dilatation, enabling the GSV to accommodate a 6–7 F filter. Therefore, VCF placement via puncture of the GSV has a theoretical and practical basis, and the feasibility is demonstrated in the current study.

In this study, as the affected lateral GSV in the DVT patient is one of the important compensatory lateral branches, the puncture is conducted on an unaffected lateral GSV. This avoids injury to the affected lateral GSV and the effect on compensatory venous backflow. During follow-up, GSV injuries caused by the puncture, including thrombus formation, valve damage and blood reflux, have not been observed. Filter placement and transcatheter thrombolysis via puncture of the GSV were performed on two patients with central type DVT. The entire procedure was completed with only one puncture wound on the superficial vein, and it was minimally invasive with a satisfactory result. However, this method still has clinical disadvantages, as the filter sheath may cause shedding of the thrombus, leading to acute pulmonary embolism.

The VCF used in this study, with a 6 F diameter, is much thinner than other commercially available products. Furthermore, this filter has advantages such as a simple releasing procedure, convenient positioning and a higher success rate of retrieval (retrievable filter) (26–30). At present, retrievable filters are more likely to be used in a clinical setting, for the following reasons: firstly, the indications for filter placement are controversial, and previously reported results of long-term efficacy and complications differ (31–34); and secondly, in younger patients with DVT, a permanent filter would not be suitable.

In the current study, VCF placement via puncture of the GSV has not provided optimal results. The injury risk of GSV puncture remains and, most notably, commercially available filters have not provided the ideal minimally invasive result. In our hospital, GSV radiofrequency laser closure surgery has been conducted on >500 patients and as such we have accumulated a large amount of experience. Puncture of the GSV on the upper medial malleolus has been conducted successfully in the majority of patients, with the placement of a 6–8 F sheath. The advantages of puncture at this position are as follows: i) The surgery is simple. For the majority of patients ultrasound positioning is not required, and a trocar may been placed under direct vision. It is possible to complete the puncture using a small guide wire (diameter, 0.018 inch). ii) As the GSV is located at the surface of the medial malleolus, it is easier to conduct the compression hemostasis, and wound oozing is easily observed. However, due to the shorter length of the filter, it is not possible to further attempt these measures at present.

In conclusion, VCF placement via percutaneous puncture of the GSV is a new filter placement method. The feasibility and safety of this method for the prevention of pulmonary embolism has been demonstrated in a small set of sample cases.

## Figures and Tables

**Table I.** Clinical and puncture characteristics.

Total (n)	12
Male	5
Female	7
Age, mean (range), years	51.5 (48–87)
DVT (n)	
Central	4
Peripheral	4
Mixed	4
GSV puncture (n)	
Left	9
Right	3

DVT, deep venous thrombus; GSV, great saphenous vein.
